# Drug development for cryptococcosis treatment: what can patents tell us?

**DOI:** 10.1590/0074-02760180391

**Published:** 2019-01-31

**Authors:** Juliana Santos-Gandelman, Alice Machado-Silva

**Affiliations:** 1 Fundação Oswaldo Cruz-Fiocruz, Instituto Nacional de Ciência e Tecnologia de Gestão da Inovação em Doenças Negligenciadas, Centro de Desenvolvimento Tecnológico em Saúde, Rio de Janeiro, RJ, Brasil; 2 Fundação Oswaldo Cruz-Fiocruz, Instituto René Rachou, Belo Horizonte, MG, Brasil

**Keywords:** cryptococcosis, Cryptococcus, cryptococcal meningitis, patent landscape

## Abstract

**BACKGROUND:**

Cryptococcosis is one of the most devastating fungal infections in humans. Despite the disease’s clinical importance, current therapy is based on limited antifungals that are either toxic, inefficient, unavailable worldwide, or that quickly lead to resistance.

**OBJECTIVES:**

The goal of this study was to provide insight into the future of cryptococcosis treatment by describing the patent scenario in this field.

**METHODS:**

We identified and analysed patent documents revealing compounds with anti-cryptococcal activity supported by experimental evidence.

**FINDINGS:**

Patenting in this field has been historically low, with an overall tendency of increase since 2012. Most applications are single filings, suggesting that they do not encompass strategic inventions requiring broad protection. Research and development essentially took place in China and the United States, which also represent the main countries of protection. Both academic and corporate institutions contributed to patenting in this field. Universities are the leading actors, with the highest patent family counts.

**CONCLUSION:**

The low number of patents in this field indicates that efforts to mitigate the unmet needs for cryptococcosis treatment remain insufficient. Without investment to drive research and innovation, patients will likely continue to face inadequate assistance. Given the current scenario characterised by poor funding and low interest for technological development, drug repurposing may be the best alternative for cryptococcosis treatment.

Cryptococcosis was recognised as a major health threat during the AIDS pandemic of the 1980s.^1^ This fungal infection, mainly caused by *Cryptococcus neoformans* (*C. neoformans*) and *Cryptococcus gattii* (*C. gattii*), is among the most lethal infectious diseases.^2^ Cryptococcosis is associated with high mortality and morbidity, globally accounting for approximately 223 000 infections and 181 000 deaths per year (estimates from 2014).^3^ ^,^ ^4^ It primarily affects immunocompromised patients, though it may also commit immunocompetent individuals. The main sites of infection are the lungs and the brain, the latter resulting in life-threatening meningitis/meningoencephalitis. Disseminated cryptococcosis is rarer, mainly occurring in HIV-infected patients, but cases in apparently immunocompetent individuals have been reported.^5^ ^,^ ^6^

Cryptococcal meningitis is the most common cause of meningitis in individuals with HIV in regions of the world with high rates of HIV infection.^7^ It remains the second most prevalent cause of death in patients with AIDS.^4^ Improvements in HIV-therapy have led to a decrease of HIV-related cryptococcal infections in countries where such therapies are available.^8^ However, with the widespread use of immunosuppression therapy, cryptococcosis is becoming increasingly common in non-HIV patients such as individuals receiving organ transplants^9^ ^,^ ^10^ or undergoing chemotherapy.^11^ In individuals who survive cryptococcal meningitis, a variety of sequalae may ensue, including focal neurologic deficits, blindness, deafness, cranial nerve palsies, and memory deficits.^11^

Despite the substantial impact of cryptococcosis, none of the standard antifungals currently used to treat this disease [amphotericin B (AmpB), flucytosine (5-FC), and fluconazole] were launched after the 1990s.^12^ ^,^ ^13^ ^,^ ^14^ These antifungals are either toxic, inefficient, unavailable worldwide, or quickly lead to resistance^15^ (see Table I). Hence, there is an urgent need for novel and improved, less toxic, more widely available and affordable treatments for this fungal disease.^21^

Although the clinical severity and unmet needs are evident, drug development for cryptococcosis treatment is hindered by a clear market failure. Given that this disease substantially afflicts low income populations, there is little investment and development interest on the part of pharmaceutical companies. In fact, out of approximately 200 organisations that completed the Global Funding of Innovation for Neglected Diseases (G-finder) annual report between 2013 and 2016, only public and philanthropic organisations (i.e., no pharmaceutical entities) reported having invested in cryptococcal meningitis drug development.^22^ ^,^ ^3^ At the same time, there are no international programs driving innovation in the area. Cryptococcal meningitis is classified among the most poorly-funded neglected diseases covered by the G-finder annual report, receiving 0.2% of global research and development (R&D) funding.

**TABLE I:** Standard antifungals for cryptococcosis treatment

	Amphotericin B	Flucytosine (5-FC)	Fluconazole
Antifungal class	Polyene	Nucleoside analogue	Azole
Year/country of first launch	1958 (US)^16^	1972 (US)^16^	1988 (UK)^16^
Mechanism of action	(i) Binds to ergosterol, disrupting fungal cell membrane (ii) induces cell death via oxidative damage^(1)^	Interferes with DNA and protein synthesis^(1)^	Inhibits cytochrome p450, interfering with ergosterol biosynthesis and cell membrane integrity^(17)^
Main advantages	High pharmacological efficacy; rare cases of resistance^18^	High pharmacological efficacy in combination with amphotericin B; available in oral formulation^18^	Low cost; oral administration; widely available^(19)^
Main drawbacks	Severe nephrotoxicity; requires intravenous administration and hospitalisation;^19^ availability and cost^(18)^	Severe hepatotoxicity;^19^ resistance (if in monotherapy);^20^ limited availability; cost^(18)^	Fungistatic (not fungicidal);^19^ Resistance^20^

To investigate the impact of this funding scarcity, Rodrigues and Albuquerque compared the number of publications in Web of Science related to cryptococcosis and other fungal diseases to that of malaria and tuberculosis - neglected tropical diseases (NTDs) that have well-stablished funding programs. Whereas 8827 and 5687 articles were published in 2017 for malaria and tuberculosis, respectively, cryptococcosis was much less investigated, only generating 213 articles.^23^ The current study aims to provide further insight into the future of cryptococcosis treatment by describing the patent scenario in this field.

## MATERIALS AND METHODS

*Search scope and strategy* - Searches were carried out between November 2017 and February 2018 using the commercial database Orbit Intelligence (Questel, Paris, France). Our search strategy targeted inventions for which the very first patent application was filed between 01/01/1995 and 31/12/2015, anywhere in the world, i.e., documents with earliest priority between these dates. We searched for documents containing the following words in their title, abstract, or claims: cryptococ*, neoformans, gattii, or torulosis. After this initial search, the following documents were selected for further analysis: (i) documents classified as A61K (preparations for medical, dental, or toilet purposes) or A61P (specific therapeutic activity of chemical compounds or medicinal preparations) by the International Patent Classification (IPC) and/or Cooperative Patent Classification (CPC) and (ii) documents including the words treat*, cure, or therap* in their title or abstract, even if not classified in the abovementioned patent classes. This last search step was an attempt to broaden our search strategy, encompassing inventions inside our scope but not classified as A61K or A61P.

*Grouping of search results into patent families* - Documents retrieved by our search were automatically grouped by Orbit into FamPat patent families. FamPat groups together patent documents that are believed to cover the same invention, e.g., different stages of an application in a given country or related applications filed in different countries. When required, these documents were automatically ungrouped into individual patent filings, i.e., FullPat records.

*Manual cleaning of search results* - Patent families were analysed individually to exclude inventions outside our search scope or not showing evidence of anti-cryptococcal activity. Inventions revealing possible drug targets, e.g., an essential fungal gene, but lacking experimental evidence of compounds with anti-cryptococcal activity were considered to fit this last exclusion criterion.

*Normalisation and de-duplication of assignees* - Assignee names were normalised using Orbit Intelligence’s assignee grouping functionality. Alternative spellings and subsidiaries were grouped under a single name when this information was known. The data was then manually cleaned to include the research institution’s name when the university’s funding agency, board of regents, or technology transfer office appeared as the assignee instead of the university’s name.

*Identification of R&D country* - As recommended by the Organization for Economic Cooperation and Development (OECD), we used inventor address information to infer where R&D took place.^19^ When inventor address and assignee address diverged, the case was analysed further for clarification. In cases where no inventor address was available for the patent family, assignee address was used instead. If no address information was available, earliest priority country was used (i.e., the country of filing of the first patent application from the respective family).

*Countries of protection* - To determine where protection is sought (i.e., countries where patents are still alive, either granted or pending), FullPat records were filtered by patent legal status and analysed by country code.

*Assignee classification -* Our revised assignees were manually classified as “Academy” (universities, research institutes, and other not-for-profit entities), “Corporate” (companies), or “Individuals” (where an individual was indicated as assignee without affiliation to any organisation). To compile assignee counts by type, assignees were only counted and classified once, even if they appeared as assignee in more than one patent family. To assess collaborations (assignee counts by type and number), assignees were classified and counted each time they were indicated as a patent family assignee, even if they had already appeared in a previous patent family.

## RESULTS

Our search strategy resulted in the retrieval of 1501 patent families. Each patent family contains one or more individual patent applications related to a single invention, corresponding, for instance, to applications filed in different countries. Patent families and individual patent filings are herein referred to as FamPat and Fullpat, respectively. The patent families retrieved by our search strategy then went through two selection filters: the first excluded inventions outside our search scope (i.e., unrelated to cryptococcosis treatment), while the second excluded inventions inside our search scope but not showing evidence of anti-cryptococcal activity. After the second filter, only 35% of inventions inside our search scope remained (i.e., 295 patent families corresponding to a total of 1525 individual patent applications/FullPat counts). These are the patent families that disclosed a compound, molecule, or extract for cryptococcosis treatment with experimental support that were first filed between January 1995 and December 2015. All of our analyses are based on this specific set of patent families.

**Fig. 1:**
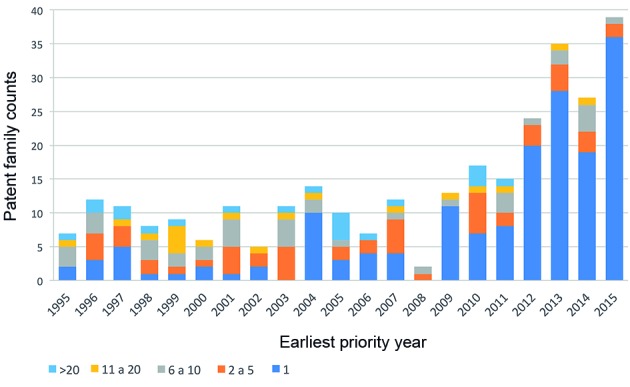
patent dynamics for cryptococcosis treatment between 1995 and 2015. The number of patent families containing experimental evidence of anti-cryptococcal activity is shown by earliest priority year, i.e., the year the first patent in the family was filed. Colours indicate the total number of individual patents in each family.

*Patenting in the field of cryptococcosis treatment has increased since 2012* - To obtain an overall picture of inventive activity related to cryptococcosis treatment, patent family counts were plotted by earliest priority year. Earliest priority year was chosen as the closest date to the invention and best indicator of inventive performance, following the OECD’s recommendations.^24^ Additionally, patent families were classified by size as an indication of investment in the protection of each invention. Our results demonstrate that patenting activity for cryptococcosis treatment has been historically low, with less than 15 patent families filed each year. However, there was an increasing trend in filings, especially from 2012 onward, that peaked in 2015. Whereas the early years of our analysis saw filings in two or more countries, the later years are characterised by single filings in individual countries. In fact, the increase in filings observed since 2012 was essentially driven by a large number of inventions for which patent protection was sought in a single country. The most impressive patent expansion was observed in 2005: 180 individual patent applications coming from 10 patent families (Fig. 1).

**Fig. 2:**
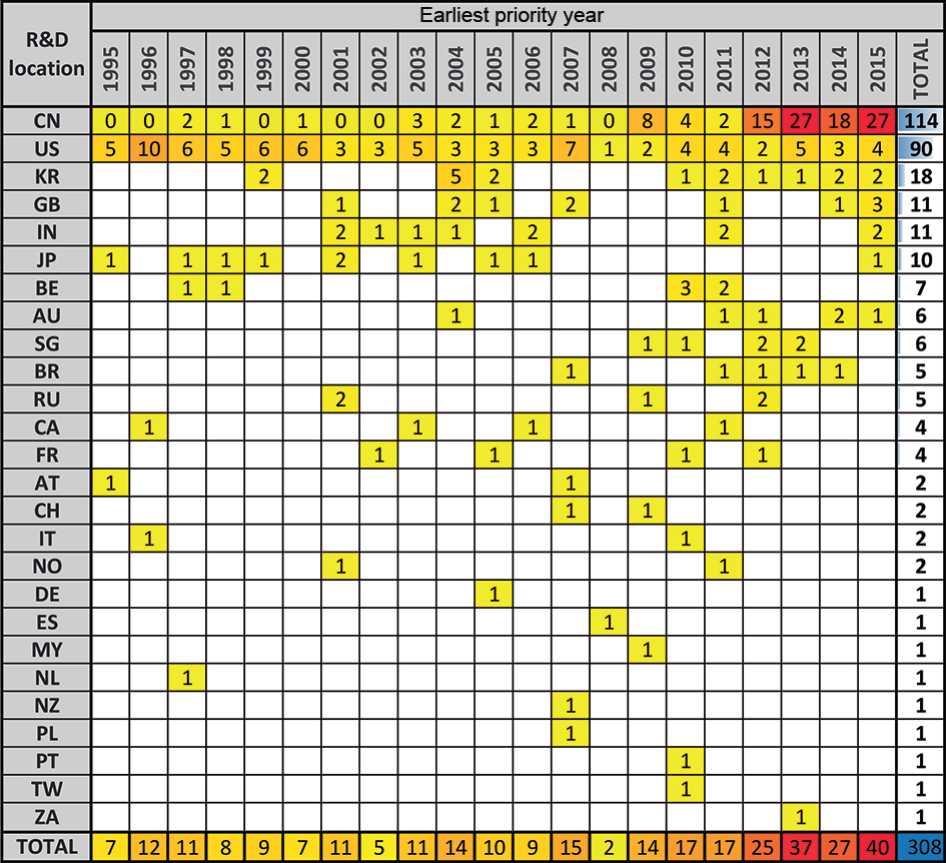
R&D origin. Patent families are classified by R&D location and earliest priority year. The warmest colours on the yellow to red scale indicate years with the highest numbers of patent applications. The total number of patents filed is shown per earliest priority year (bottom line) and for each R&D location (column on the right). Country codes: China (CN), United States (US), South Korea (KR), Great Britain (GB), India (IN), Japan (JP), Belgium (BE), Australia (AU), Singapore (SG), Brazil (BR), Russian Federation (RU), Canada (CA), France (FR), Austria (AT), Switzerland (CH), Italy (IT), Norway (NO), Germany (DE), Spain (ES), Malaysia (MY), Netherlands (NL), New Zealand (NZ), Poland (PL), Portugal (PT), Taiwan (TW), and South Africa (ZA).

*Applications from Chinese residents were the main driver of patenting growth* - To infer where R&D activity took place and further investigate the above-mentioned increasing trend in patent filings, our data was analysed by inventor country of residence as recommended by the OECD^24^. This analysis indicated that R&D activity took place mainly in China (CN) and the United States (US) (66% of patent families came from residents of these two countries). Another 6% of patent families were filed by residents of South Korea (KR), the United Kingdom (GB), India (IN), and Japan (JP). Patent filings by Chinese residents significantly increased from 2012 onward, driving the overall growth in patent filings. In fact, 76% of filings by Chinese residents took place after 2011. To the contrary, a slightly decreasing trend in filings by US residents was observed (58% of such filings occurred in the first ten years of our analysis and 42% in the last ten years). It should be noted that in certain cases, R&D was carried out in more than one country. Therefore, the total family count for this analysis was 308 and not 295 (Fig. 2).

**Fig. 3:**
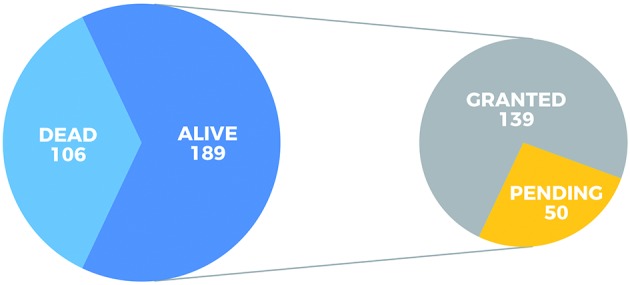
patent family status. Patent families are classified by status. Families are considered alive if they have at least one member still in force. When the live family contains at least one granted patent, the whole family is classified as granted. Otherwise the family is regarded as pending, indicating applications belonging to this family are still under review by the respective national patent offices.

**Fig. 4:**
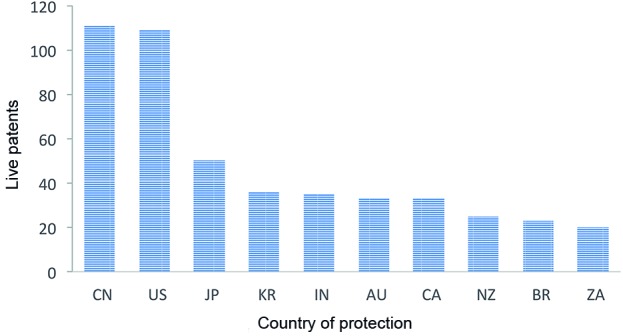
countries of protection for cryptococcosis treatment. The number of live individual patent counts (FullPat) is shown by country of filing, indicating where protection is sought. Only countries with 20 or more live patents are represented. Country codes: China (CN), United States (US), Japan (JP), South Korea (KR), India (IN), Australia (AU), Canada (CA), New Zealand (NZ), Brazil (BR), and South Africa (ZA).

*Most patent families are alive and were filed in China and the US* - Patent counts include applications that are alive, either pending examination or granted, but also applications that are already dead, i.e., abandoned by the assignee, expired, or revoked. Hence, patent legal status was analysed to ascertain how many of these patent families currently protect inventions or have the potential to protect them. Our results showed that most of the 295 patent families (64%) are alive (i.e., they have at least one live member, either granted or pending). From these live families, 74% contain at least one patent in force (i.e., granted), whereas 26% consist of pending applications (Fig. 3). Assignees usually file patents in countries deemed strategic for their inventions - those with the most promising markets for the invention, in economically important regions, in the country where the assignee is actually based or the home country of potential licensors, among others. Our analysis showed that most of the patents were filed in China (CN) and the United States (US). Japan (JP), Australia (AU), Canada (CA), India (IN), South Korea (KR), Brazil (BR), New Zealand (NZ), and South Africa (ZA) followed suit, all with 20 or more live patents. Only countries with 20 or more live patents were included in the image shown in Fig. 4.

**Fig. 5:**
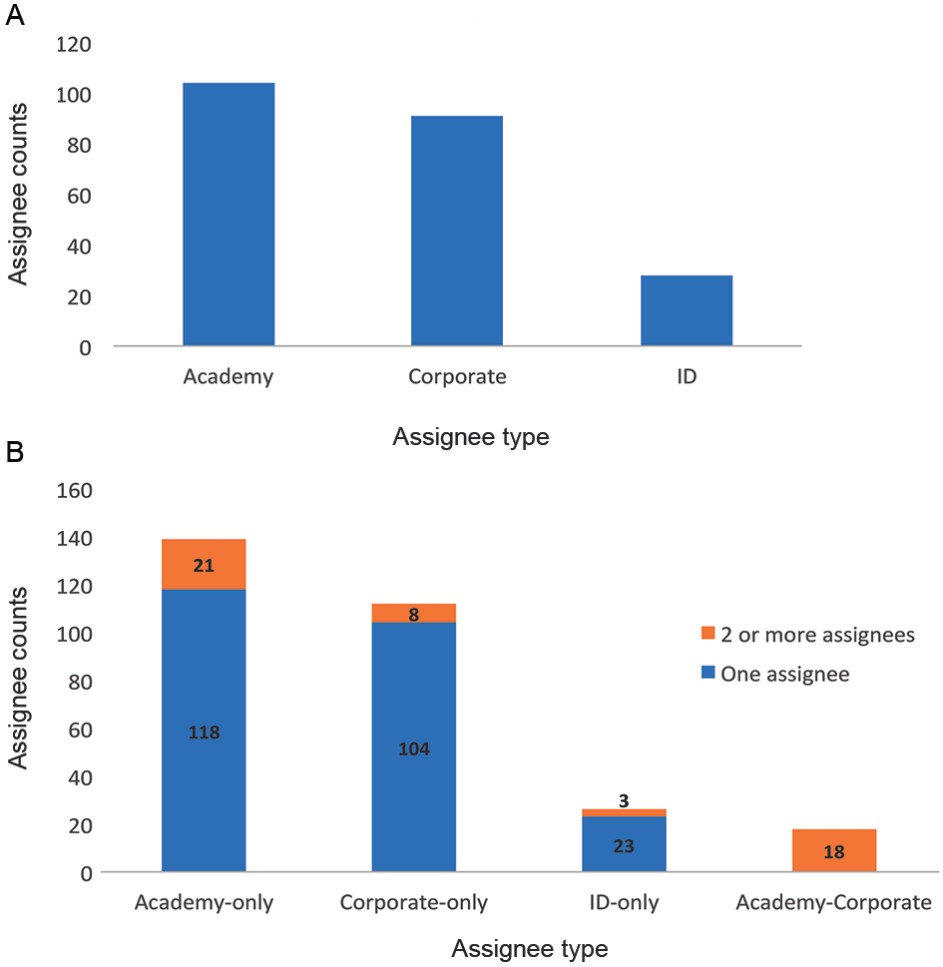
assignee type and collaborations. (A) Classification of assignee counts by assignee type. Assignees were classified as “Academy” (universities, research institutes, and other not-for-profit entities), “Corporate” (companies), and “ID” (individual without affiliation to any organisation). (B) Classification of patent families by assignee type and number. Assignees were classified as “academy-only,” “corporate-only,” “ID-only,” or “academy-corporate.” Patent families with a single assignee are represented in blue, whereas those with two or more assignees are depicted in orange.

*Both academic institutions and companies contributed to patenting directed at cryptococcosis treatment, albeit with low levels of collaboration* - To provide information on the institutions behind the inventive activity directed at cryptococcosis treatment, assignees were classified as “Academy” (universities, research institutes, and not-for-profit organisations), “Corporate” (companies), or “Individuals” (individuals without institutional affiliations). As shown in Fig. 5, 47% of our patent family assignees were classified as “Academy”, 41% as “Corporate” and 12% as “Individuals” (Fig. 5A). Almost half of the patent families (47%) came from the academic sector exclusively, 38% were corporate-only patents, and 9% were assigned to individuals. A low percentage of these patent families had more than one assignee (17%). Of these, 42% were co-assigned by the academic sector, 36% by the academy and corporations, 16% by corporations, and 6% by individuals (Fig. 5B).

**Fig. 6:**
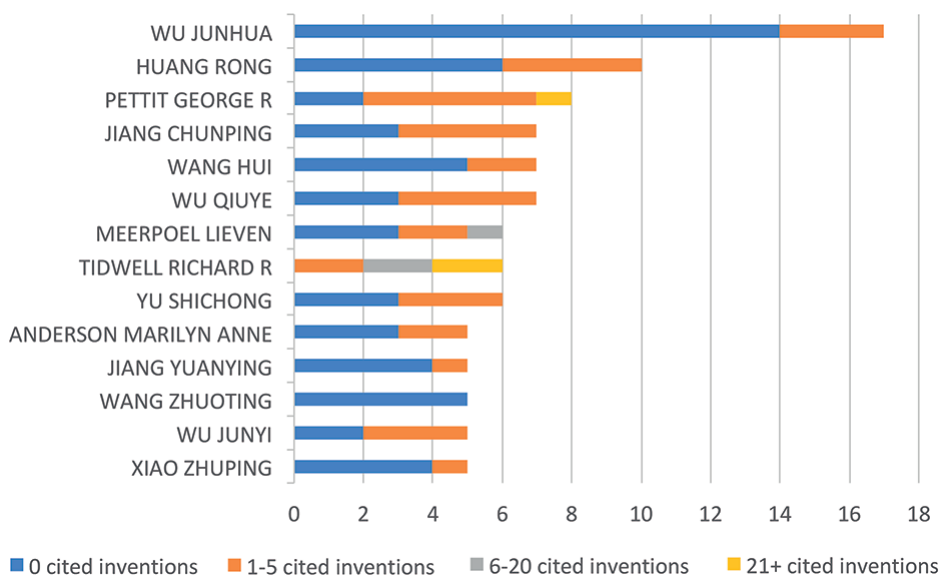
main inventors. Researchers are ranked by the frequency which they appear as inventors (calculated at the patent family level). Only individuals appearing in five or more patent families are represented. Colours indicate the number of times a patent family is cited in subsequent patent documents from a different assignee (non-self forward citations).

**Fig. 7:**
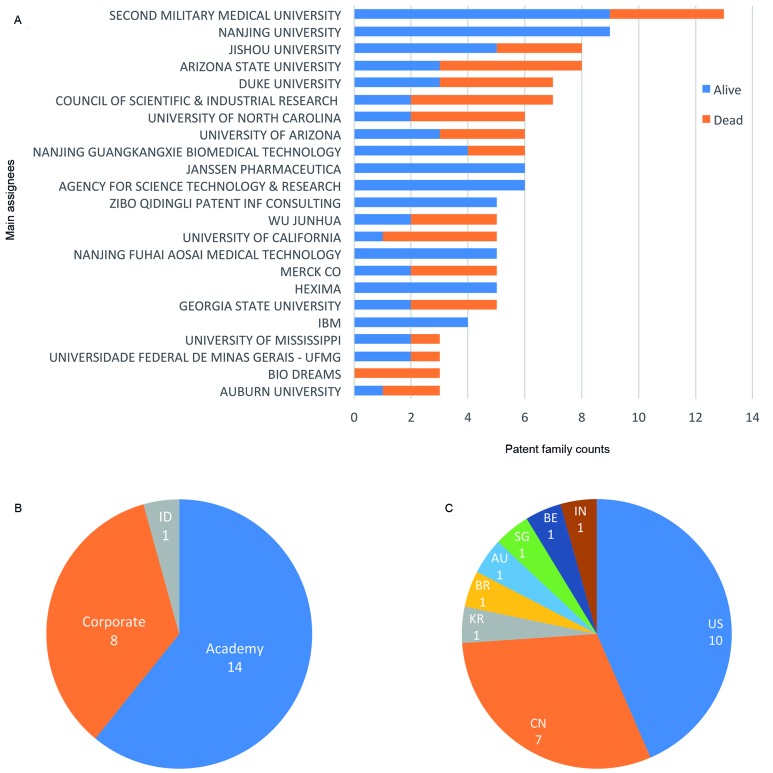
main assignees. Institutions are ranked by their frequency of appearance as assignee. Only owners of three or more patent families are represented. (A) Number of patent families by assignee. Live families are shown in blue, whereas dead families are represented in orange. Families are considered alive if they have at least one member in force. (B) Classification of main assignees by assignee type. Assignees were classified as “Academy” (universities, research institutes, and other not-for-profit entities), “Corporate” (companies), and “ID” (individual without affiliation to any organisation). (C) Classification of main assignees by assignee location. Country codes: United States (US), China (CN), South Korea (KR), Brazil (BR), Australia (AU), Singapore (SG), Belgium (BE), India (IN).

*Top-cited inventors and best-ranked assignees are from the academic sector* - Patent landscapes can offer insights into the main players (inventors or institutions) in specific technological fields. This information can be useful in the identification of both competitors and potential collaborators for future R&D. Fig. 6 shows the inventors with the most patent family counts in the field of cryptococcosis treatment. Only inventors with five or more patent families are represented. Non-self forward citations are included for each of the main inventors’ patent families as a measure of invention impact. This is a common metric in patent analysis that represents the number of times patents from an assignee are cited in applications from a different assignee (calculated at the family level). Lieven Meerpoel (Janssen Pharmaceutica, Beerse, Belgium), Richard Tidwell (University of North Carolina, Chapel Hill, NC, USA), and George Pettit (Arizona State University, Phoenix, AZ, USA) are the only inventors with six or more non-self forward citations. The latter two are inventors in patent families that received more than 21 non self-citations. The leading institutions in this field (here defined as owners of three or more patent families) are represented in Fig. 7. Apart from the company Bio Dreams (Daejeon, South Korea), all have at least one live patent family in their portfolio (patent families having at least one live member are considered alive) (Fig. 7A). The best-ranked assignees are all universities and 61% of assignees owning three or more patent families are from universities or governmental institutions (Fig. 7B). Regarding geographic location, most are institutions based in the US (43%) and China (30%) (Fig. 7C). It is noteworthy that the larger patent families, with 20 or more individual patent filings, usually presented *in vivo* evidence against *Candida* sp. and *Aspergillus* sp. and only *in vitro* tests against *Cryptococcus* sp. (data not shown).

*Most new entrants in this field are institutions and companies based in China* - Given that our patent analysis covered 20 years, some assignees may have been active in the early years of our analysis but have since lost interest in this field. Indeed, an analysis of the timeline of patent filings by our top assignees showed that 17% of them did not file any patents in the last ten years of our analysis (2006-2015), whereas 26% were new entrants that only filed patents during this later period. The majority of new entrants (83%) were universities and companies based in China. Out of the top assignees that have been inactive in the last 10 years, most (75%) are US-based entities (Table II).

**TABLE II:** New entrants and inactive players (2006-2015)

New entrants	Inactive players
Nanjing Fuhai Aosai Med Tech (CN)	Biodreams (KR)
Nanjing Guangkangxie Biomed Tech (CN)	Georgia State University (US)
Nanjing University (CN)	Merck Co (US)
Second Military Medical University (CN)	University of North Carolina (US)
Universidade Federal de Minas Gerais (BR)	
Zibo Qidingly Patent Inf Consulting (CN)	

## DISCUSSION

Patent landscapes make use of information included in patent literature to provide an overview of the patenting scenario in a given technological field. This information can be used to inform policy discussions and business decisions, as well as to guide strategic research planning, helping to identify the main players, new entrants, R&D locations, and countries of protection, among others.

An important limitation of patent landscapes is that patent applications are drafted to maximise protection, making broad claims that are not necessarily experimentally confirmed. To circumvent this limitation, only documents containing evidence of anti-cryptococcal activity were added to our analysis. In the absence of such a filter, we would obtain a picture of all patents that claim to disclose a compound with anti-cryptococcal activity, even if *Cryptococcus* was included in the patent document as another fungus among many in the middle of a long list of microorganisms, for which no evidence of activity is available. Indeed, only 35% of patents retrieved in our search and inside our search scope (cryptococcosis treatment) actually contained evidence of anti-cryptococcal activity. Hence, for the current landscape to more closely reflect patenting in this specific field, the use of experimental evidence as a selection criterion is crucial. A drawback of this approach is that evidence may become available after the patent is filed. In these cases, patents disclosing such compounds would be missed. Nevertheless, patent landscapes are snapshots of the patenting situation at the time of data analysis and not a follow up of further developments that may have occurred after filing. In any case, we conclude that the overestimation resulting from the inclusion of all documents regardless of experimental evidence would be much more prejudicial than the possible underestimation resulting from the use of an experimental evidence filter.

Our results indicate little interest in drug development for cryptococcosis. This is suggested by the following findings: (i) the number of patents that actually present some evidence of activity against *Cryptococcus* sp. is quite low, despite the increasing trend since 2012; and (ii) none of the top inventors from our patent search list cryptococcosis as their main line of research, although some focus on the development of antibacterial and antifungal compounds and on other NTDs (data not shown). These findings are also supported by previously published analyses of this same patent collection,^25^ which indicate the following: (i) cryptococcosis had a secondary position in most of these patents; (ii) experimental evidence against *Cryptococcus* sp. was usually very preliminary, consisting mostly of MIC (minimal inhibitory concentration) tests; and (iii) less than 5% of the companies appearing as assignees in our selected patent documents actually included cryptococcosis treatment in their publicly available pipeline. Such low interest is not surprising, given that R&D funding for drug development targeting cryptococcosis is very scarce despite the clinical importance of this fungal infection.^15^

The increase in filings since 2012 could indicate hope for improvement in the treatment of cryptococcosis. However, these patent families are essentially formed by individual applications. This suggests they do not encompass very strategic inventions that would require broad protection. In the case of blockbuster drugs, for instance, patents are filed in several countries and a single patent family may contain hundreds of patents. Hopefully in the years to come, this increasing trend in filings will be followed by a similar growth in patent family size.

Our results seem to suggest decreasing interest in the field from US residents, given the slight decline in the respective patent filings since 2005 and the fact that most inactive players in this same period are US-based institutions. The rise in patent filings since 2012 was mainly driven by Chinese applications and most new entrants in this field are also Chinese, revealing important contributions of Chinese institutions to patent filings in this area. Indeed, this may be the result of an intensification in Chinese R&D efforts aimed at cryptococcosis treatment. However, patenting incentives introduced by China’s National Patent Development Strategy (2011-2020) may also have fueled patent numbers. The strategy introduced measures to enhance China’s intellectual property system by encouraging local individuals, institutions, and companies to pursue intellectual property protection domestically and abroad. It included quantitative patent-per-capita targets to be reached by the end of specific years (set at the national and provincial/municipal levels) and government incentives for filing them, including subsidies to cover patent costs. This resulted in an upsurge in patent applications in China by Chinese residents, not exclusively resulting from increased R&D. Chinese patent subsidies also encouraged the following strategies: (i) repeated patent applications filed for the same invention; (ii) splitting technological development into smaller inventions to boost the number of applications; (iii) filings for inventions already disclosed; and (iv) filing applications only to meet patent targets.^26^ The strategy of splitting inventions was identified in approximately 11% of the Chinese residents’ patents retrieved in our analysis (data not shown).

Our assessment of countries wherein patent protection is most often sought showed China and the US in the top positions. As these are also the main R&D countries, this result appears to reflect the usual bias for filing domestic applications (applicants tend to file patents in their home country). South Africa was the only African country with 20 or more live patents.^4^ In fact, only a total of 35 patents are alive on the African continent, 11 of which were filed via regional treaties (data not shown). Considering that sub-Saharan Africa experiences the highest disease burden, one would expect a higher number of patent filings in this region. This could be a reflection of sub-Saharan Africa’s insufficient economic returns for pharmaceuticals.

An analysis of all patent assignees indicated that both the academic sector and companies contributed to patent filings in this field. The academic sector seems to have a slightly more prominent role, given that the top assignees and most-cited inventors are from this classification, as are 64% of the patents that actually disclose *in vivo* evidence against *Cryptococcus* sp.^25^ This finding is in agreement with the idea that currently both the academic sector and pharmaceutical companies play important roles in drug development for NTDs. According to a recent study, the public sector and philanthropic organisations sponsored the largest share of clinical trials for neglected diseases registered at clinicaltrials.gov between 2005 and 2015. Nevertheless, the majority of phase III clinical trials, which are the most costly and time consuming, had pharmaceutical companies as their main sponsors.^27^ Furthermore, most large pharmaceutical companies currently have research and development units focused on NTDs.^28^

In view of the inadequate funding directed at cryptococcosis^15^ ^,^ ^22^ ^,^ ^23^ and its respective impact on scientific production^23^ and considering the scenario for technological development described in this study, drug repurposing may be the best alternative to fast track drugs for cryptococcosis treatment. This strategy offers attractive benefits for pharmaceutical companies, in that it considerably reduces the resources required for developing therapeutic solutions for any given disease.^29^ In fact, sertraline hydrochloride and tamoxifen are promising compounds for repurposing already in the clinical trial stage for cryptococcal meningitis (clinical.trial.gov identifier NCT01802385 and NCT03112031, respectively). Both clinical trials are sponsored by the academic sector and involve academic collaborations.

Given the lack of collaboration in the area, investing in collaborative work may also drive innovation in this field, engaging experts to work together toward a common goal sharing R&D risks and costs. More specifically, academia-pharma partnerships have the added benefit of maximising the strengths of the respective partners, integrating expertise in the technical field with knowhow to translate research findings into drugs.^30^ Such partnerships may materialise through a variety of arrangements, including product development partnerships (PDPs), open innovation, public-private consortia, and joint ownership of laboratories. In fact, PDPs, which bridge public and private research entities with philanthropic and public funding, were the primary sponsor for 46% and 56% of new neglected disease drug approvals in the periods from 2000 to 2008 and 2009 to 2013, respectively.^31^

Despite the many advances brought about by collaborative R&D efforts, the number of approved drugs in recent decades is far from ideal, and many challenges still exist for NTD drug development.^31^ ^,^ ^32^ ^,^ ^33^ In the specific case of cryptococcosis, increased funding is imperative to drive both research and innovation. The inclusion of cryptococcosis as an NTD could be an important step in this direction: (i) by raising awareness to the fact that cryptococcosis ranks among the most poorly funded diseases in the world;^17^ (ii) by allowing the academic sector and corporations to benefit from global NTD funds; (iii) by incentivising the establishment of governmental, philanthropic, and institutional funding programs directed to this specific disease; and (iv) by ensuring that afflicted populations will benefit from global initiatives to reduce NTD burden such as the London Declaration on NTDs. If no action is taken, patients will most likely continue to receive inadequate assistance and effective treatment will remain unavailable.
